# Importance of periodontal phenotype in periodontics and restorative dentistry: a systematic review

**DOI:** 10.1186/s12903-023-03777-3

**Published:** 2024-01-08

**Authors:** Mohan Kumar P, Raghavendra Reddy Nagate, Saurabh Chaturvedi, Manae Musa Musleh Al-Ahmari, Mohammed A. Al-Qarni, Shankar T Gokhale, Abdul Razzaq Ahmed, Ahmed Al Bariqi, Marco Cicciù, Giuseppe Minervini

**Affiliations:** 1 Department of Periodontics and Implantology, Vishnu Dental College, West Godavari, Vishnupur, Bhimavaram, 534202 Andhra Pradesh India; 2 Department of Periodontics and Community Dental Sciences, College of Dentistry, King Khalid University, Abha, Saudi Arabia; 3 Department of Prosthetic Dentistry, College of Dentistry, King Khalid University, Abha, Saudi Arabia; 4 Consultant in Restorative Dentistry, College of Dentistry, King Khalid University, P.O.Box 3263, Abha, 61471 Saudi Arabia; 5 Department of Biomedical and Surgical and Biomedical Sciences, Catania University, Catania, 95123 Italy; 6 Saveetha Dental College and Hospitals, Saveetha Institute of Medical and Technical Sciences (SIMATS), Saveetha University, Chennai, Tamil Nadu, India; 7 Multidisciplinary Department of Medical-Surgical and Dental Specialties, University of Campania “Luigi Vanvitelli”, Caserta, 81100 Italy

**Keywords:** Periodontal phenotype, Periodontics, Restorative dentistry

## Abstract

**Background:**

Periodontal phenotype is regarded to be one of the key factors influencing the efficacy of restorative therapies in dental practice. The objective of the systematic review was to explore the importance of thin and thick periodontal phenotypes and how they affect the outcome of periodontal and restorative therapies by looking at a number of academic publications from various online databases.

**Methods:**

Following the PRISMA guidelines (Preferred Reporting Items for Systematic Review standards), relevant data will be searched and retrieved from three significant scientific databases, including PubMed, EBSCO, and Scopus. The articles with full texts that matched the keywords and published in English between 2018 and 2023 were taken into consideration.

**Results:**

The majorities of these articles were based on the type of periodontal phenotype and their impact on periodontal and restorative treatment outcomes were selected. The initial search yielded a total of 530 articles. Only 273 were relevant to the review’s objectives, and these were considered for determining eligibility. Only 20 publications were eligible for analysis.

**Conclusion:**

Understanding these anatomical aspects of periodontal phenotype is crucial to both periodontology and restorative dentistry. The clinical outcome of restorative, prosthetic, orthodontic, surgical, and periodontal therapies is determined in large part by the periodontal phenotype, which also plays a significant role in clinical failure or success in dental treatments.

**Trial registration:**

This study protocol registered with the International Prospective Register of Systematic Reviews (PROSPERO) dated 16th June 2023 with the registration ID CRD42023432568.

## Introduction

Periodontal phenotypes are classified based on three essential features. These three factors are bone morphotype, keratinized tissue width, and gingival thickness. Each of these elements is essential to maintaining periodontal health [1, 2].

The term “gingival phenotype” refers to the morphological characteristics of the gingiva and the periodontium, while the terms “periodontal biotype, “periodontal morphotype, “gingival morphotype,” and “gingival phenotype” relate to the variations in the thickness of gingiva (GT) and width of keratinized tissue (KTW). The bone morphotype, keratinized tissue, and gingival thickness make up the periodontal phenotype. The gingival phenotype is made up of the latter two elements, which are located beneath the bone [3–6].

Gingival thickness, KTW and bone morphotype were three important parameters used to categorize biotypes and they were important in development or progression of mucogingival defects [7–9]. “Thin-scalloped, “thick-scalloped,” and “thick-flat” periodontal biotypes are the three types of periodontal biotypes that are taken into consideration in the World Workshop on the Classification of Periodontal and Peri-Implant Diseases and Conditions in 2017 [10–12].

The final aesthetic outcome of periodontal and restorative treatment methods depends on a variety of factors. The evaluation of the surrounding soft and hard tissues, which will be crucial to the success of periodontal and restorative therapies, is the most crucial of them [13–15].

Although gingival and bone thicknesses are known to influence treatment outcomes, various periodontal phenotypes may respond variably to inflammatory, surgical, and restoration techniques. After surgical procedures, poor treatment results are linked to a thin periodontal phenotype. In patients with thin GT, extra operations are typically required, whereas in people with thicker tissues, a straightforward technique can be used [16–19].

Prior to beginning the restorative treatment, it is crucial to ascertain the tissue biotype. The thickness of the gingival and bone tissues influences treatment outcomes, presumably due to changes in blood supply to the underneath bone and vulnerability to resorption [20–22].

Recession is more likely to occur in individuals with thin tissue and limited gingival width. Phenotype modification therapy (PhMT) may be beneficial for patients with thin gingival tissue and mucogingival abnormalities. The amount of mucosal recession surrounding implants may be partially reduced through surgically changing the phenotypic of the peri-implant soft tissue from thin to thick. PhMT may improve periodontal health in orthodontic patients, as well as lessen problems, improve stability, and speed up the course of orthodontic treatment [23, 24].

Dental professionals must have a sufficient understanding of current key changes in the periodontal phenotype in order to focus knowledge towards the best possible early diagnosis of patients. Several methods for improving or supplementing KT or GT have been suggested. However, it is currently unknown whether phenotype-modifying therapy (PMT) affects long-term periodontal and restoration treatment results [25, 26].

This systematic research set out to investigate the significance of thick and thin periodontal phenotypes for preserving periodontal health, especially prior to undergoing extensive restorative procedures.

## Methods

### Study design and setting

By adhering to PRISMA (Preferred Reporting Items for Systematic Review and Meta-Analysis), this systematic review concentrated on determining the importance of thin and thick periodontal phenotype in restorative and periodontal procedures. The purpose of this systematic review was to provide a comprehensive overview of the role and impact of periodontal phenotype on dental treatment outcomes.

This review was conducted from June 2023 to August 2023. The review covered publications from 2018 to 2023. This study’s analysis was conducted using JiraTM data analysis software. The Critical Appraisal Skills Programme (CASP) checklists were used to independently evaluate the study’s quality. Two distinct observers meticulously extracted the final data and reported their work. As indicated by the Kappa score of 0.75, there was good inter-observer agreement. The Boolean operator’s AND/OR/NOT was employed in PubMed to either restrict or expand the search to include all potential articles.

The research question in this study was “How the thin and thick periodontal phenotype affected the periodontal and restorative treatment outcome?”. This research question is translated using PICO which consists of Population, Intervention, Comparison, and Outcome.

PICO Search: P- Research on human adults; I- Research both with and without intervention; C- Groups of sites with thin or thick periodontal phenotype were included in the research. O- Research having data on gingival or periodontal outcome parameters were included.

### Literature search protocol

The search for the articles was done by three reviewers independently. Publications of interest within the scope of this focused systematic review were searched in the electronic database PubMed, EBSCO, and Scopus.

### Eligibility criteria for study

In this systematic review, research publications in English that met the following criteria were included: randomized controlled trials evaluating and comparing the importance of periodontal phenotype in restorative and periodontologyy were included, controlled clinical trials assessing the impact of periodontal phenotype on restorative and periodontal treatment outcomes, and any original research papers, case studies, and systematic reviews on the periodontal phenotypes both in restorative and periodontology were included. Research articles written in languages other than English, studies that don’t adhere to the objectives of the review’s research, technical remarks, short communications, editorial letters, and mini-reviews were not included.

This study protocol registered with the International Prospective Register of Systematic Reviews (PROSPERO) dated 16th June 2023 with the registration ID CRD42023432568. Based on the research publications that were available and our understanding of the subject, we came up with a list of keywords. The significance of the terms “periodontal phenotype”, “gingival phenotype”, and “bone morphotype” in periodontal and restorative treatment techniques was investigated.

The following terms used in the PubMed, EBSCO, and Scopus databases:

“phenotype“[All Fields]) AND (“treatment outcome“[MeSH Terms] OR (“treatment“[All Fields] AND “outcome“[All Fields]) OR “treatment outcome“[All Fields])

periodontal[All Fields] AND (“phenotype“[MeSH Terms] OR “phenotype“[All Fields]) AND periodontal[All Fields] AND (“treatment outcome“[MeSH Terms] OR (“treatment“[All Fields] AND “outcome“[All Fields]) OR “treatment outcome“[All Fields])

periodontal [All Fields] AND (“phenotype“[MeSH Terms] OR “phenotype“[All Fields]) AND restorative[All Fields] AND (“treatment outcome“[MeSH Terms] OR (“treatment“[All Fields] AND “outcome“[All Fields]) OR “treatment outcome“[All Fields]).

The Rayyan website was used for the research selection procedure. Results of searches from other databases that are duplicates will not be included. Inappropriate abstracts and titles will also be disregarded. The collected studies will be examined comprehensively to ensure they satisfy the predetermined inclusion and exclusion standards. They will then be evaluated for potential bias and incorporated into the qualitative synthesis (Systematic Review).

## Results

### Research identification and selection

The search strategy employed for this systematic review aimed to locate research publications from 2018 to 2023 that are pertinent to answering the research issues associated with the review’s objectives. The date last searched was August 2023. As part of the plan, the search field for the use of role of periodontal phenotypes on restorative and periodontal treatment outcomes was defined.

**Fig. 1:**
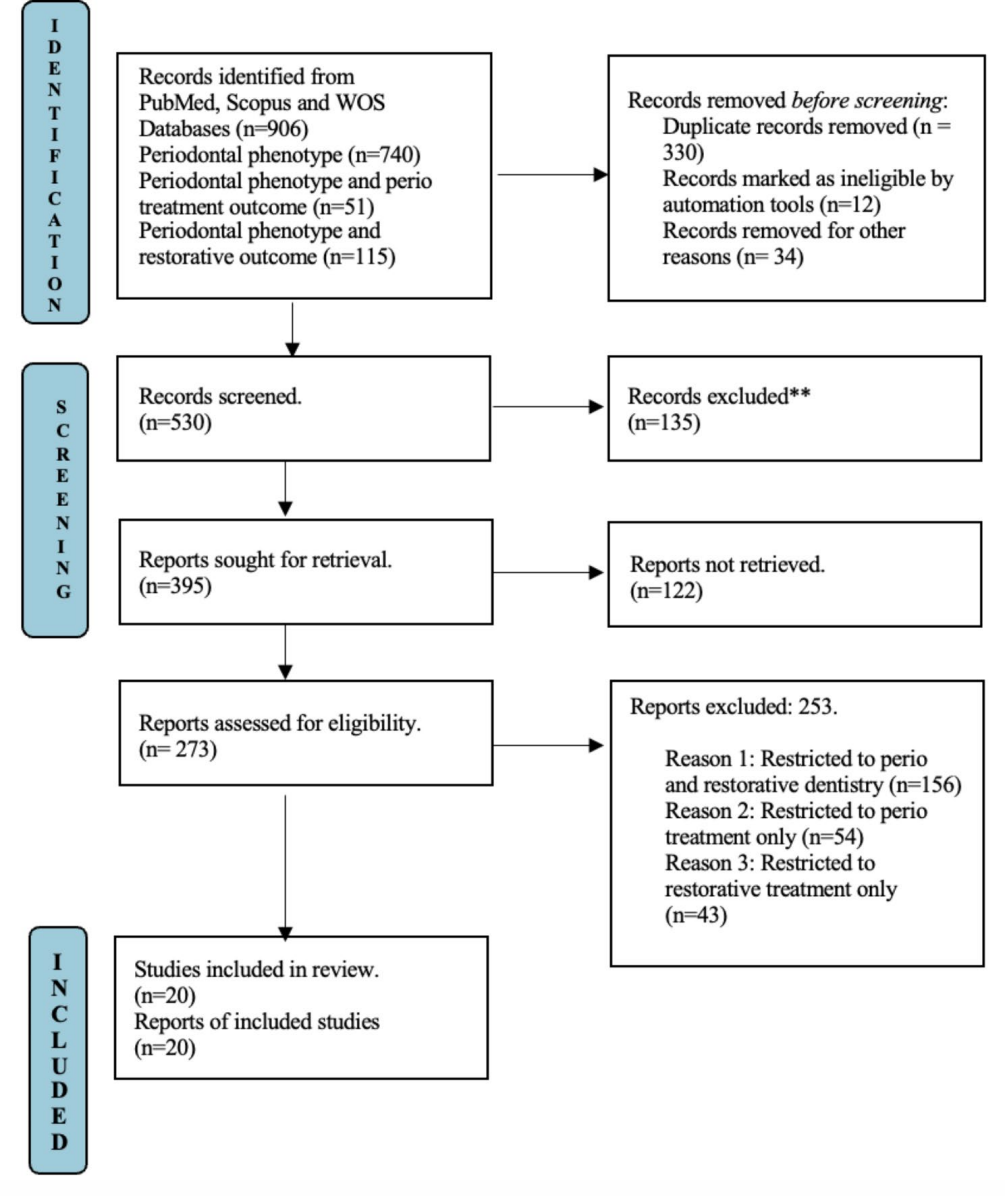
Demonstrates flow diagram of the study selection process as indicated by the PRISMA (Preferred Reporting Items for Systematic Review and Meta-analysis)

The PRISMA (Preferred Reporting Items for Systematic Reviews and Meta-Analyses) standards were used when identifying research. Figure 1 provides a flow chart. The first step in the research identification process was to search three internet databases: PubMed, EBSCO, and Scopus.

The data extraction was done by two examiners and the method followed for data extracted was based on the public sources like journal articles, clinical trials that give useful information about methods and results of included articles. The collected data was used to develop outlines of tables and figures that will appear in the review to facilitate the design of data collection forms.

A quick keyword search produced a total of 906 results. Only 530 publications were left after the initial screening to be examined in accordance with the objectives of the systematic review. Only 395 papers were sought for retrieval, of which 273 were relevant to the review’s objectives or criteria, and these were considered for determining eligibility. From all the studies that were eligible, only 20 publications that provided comparative and systematic reviews on periodontal phenotypes and their impact on periodontal and restorative treatment outcomes were chosen. (Fig. 1) The included research papers and reviews are summarized with concluding remarks based on their study purpose and significance. Table 1 lists the features of the research papers considered in this systematic review.

**Table 1:** Qualitative Synthesis Results

Sl. No	Citation No.	Type of study	Kind of Clinical trial	Number of Participants	Intervention	Outcome measures and summary
1	[16]	Review	A Literature Review	-	Gingival health	Subjects with thin and narrow gingiva tend to have more gingival recession compared with those with thick and wide gingiva.
2	[17]	Review	A Systematic review	-	Graft material	It was observed that any graft material was able to significantly enhance gingival thickness (GT).
3	[23]	Review	A Meta- regression analysis	-	Periodontal flap technique	Soft tissue thickness (STT) plays a limited role in predicting root coverage across all approaches; when flaps are performed with no graft, the effect of STT is most critical.
4	[24]	An original research	A randomized controlled clinical trial	30	Connective tissue graft	Both procedures were effective for root coverage at single RT1 recession with previously restored cement-enamel junction (CEJ). Adding a connective tissue graft (CTG) under CAF should be considered for Rec with thin gingival phenotype.
5	[25]	Case report	Case report	1	Periodontal Accelerated Osteogenic Orthodontics	In patients with dentofacial deformities and a thin periodontal phenotype, multi-disciplinary treatment that includes periodontal accelerated osteogenic orthodontics (PAOO) could be effective, and could improve both the quality and safety of orthodontic-orthognathic therapy.
6	[15]	A Review	A cluster analysis	-	Sagittal craniofacial profile	A moderate correlation was found between mandibular gingival thickness and the sagittal craniofacial profile. Patients with a concave craniofacial profile had a smaller keratinized gingiva width and gingival thickness in the aesthetic zone.
7	[28]	A Review	A Review	-	Allogenous dermal matrix	Allogenous dermal matrix (ADM) is a viable option for soft tissue augmentation, as well as for treatment approaches involving buccal bone gain.
8	[29]	An original research	A randomized controlled clinical trial	60	Sub-epithelial connective tissue graft, Leukocyte- and platelet-rich fibrin membrane	All the three surgical techniques for root coverage: the coronally advanced flap (CAF), with a sub-epithelial connective tissue graft (SCTG) or with leukocyte- and platelet-rich fibrin (L-PRF) membranes produced significant gingival recession (GR) reduction and clinical attachment level (CAL) gain
9	[31]	An original research	A randomized controlled clinical trial	20	Mini-Five Gracey Curettes	The use of Mini-Five Gracey Curettes (MFC) resulted in a greater pocket depth (PD) reduction and lower rate of recession depth (RD) in the short term.
10	[32]	An original research	A multicenter inter- and intra-examiner agreement study	28	2018 Classification of Gingival Recession Defects and Gingival Phenotype	The 2018 Classification of Gingival Recession Defects and Gingival Phenotype is clinically reproducible within the examiners, and when the variables forming the matrix are analyzed individually.
11	[33]	An original research	A randomized controlled clinical trial	50	Modified coronally advanced tunnel technique	Both the modified coronally advanced tunnel technique (MCAT) and free gingival grafts (FGG) were successful in terms of gingival phenotype modification in the anterior mandible.
12	[39]	A Review	A narrative review	-	Periodontal outcomes	Periodontal diagnostic criteria should be thoroughly reviewed before fixed restorative treatments are planned and executed.
13	[40]	A Review	A consensus statement	-	Modification of gingiva	Patients with thin gingival tissue and mucogingival defects may benefit from phenotype modification therapy (PhMT-s) intervention and may require a secondary procedure to achieve optimal outcomes.
14	[42]	An original research	A comparative cohort study	28	Soft tissue phenotype modification	Soft tissue phenotype modification, either pouch roll or modified roll technique, during uncovering surgery resulted in favorable clinical outcomes.
15	[43]	An original research	A retrospective study	111	Connective tissue grafting	Surgical modification of peri-implant soft tissue phenotype via phenotype modification therapy (PhMT-s) may decrease the amount of mucosal recession (MR).
16	[45]	An original research	Description of surgical technique	1	Omega roll envelope flap	The omega roll envelope flap technique has shown advantages as maximizes the amount of connective tissue that can be rolled within the buccal flap.
17	[46]	A case series	A prospective case series	10	Microsurgical roll-in-envelope flap	In presence of sufficient periimplant supporting tissues and when indicated, the roll-in-envelope flap (RIE) seems to yield superior outcomes reducing pain/discomfort compared to connective tissue grafts.
18	[49]	An original research	A randomized controlled clinical trial	26	Zirconia abutments	Zirconia abutments exhibited better results than titanium abutments in terms of the peri-implant tissues. Moreover, in those with a thin phenotype, zirconia provided improved gingival esthetics.
19	[50]	A case series	A Retrospective Case Series	14	Vestibular Incision Subperiosteal Tunnel Access	Treatment of multiple gingival recession defects with vestibular incision subperiosteal tunnel access (VISTA) and subepithelial connective tissue grafts (SCTG) led to stable gingival thickness gains and shows promise as a strategy for phenotype modification therapy.
20	[51]	An original research	A Controlled Clinical Trial	41	Connective tissue graft	There are no significant differences in the outcomes of immediate implant placement for patients with different soft tissue phenotypes.

### Risk of bias assessment

**Fig. 2:**
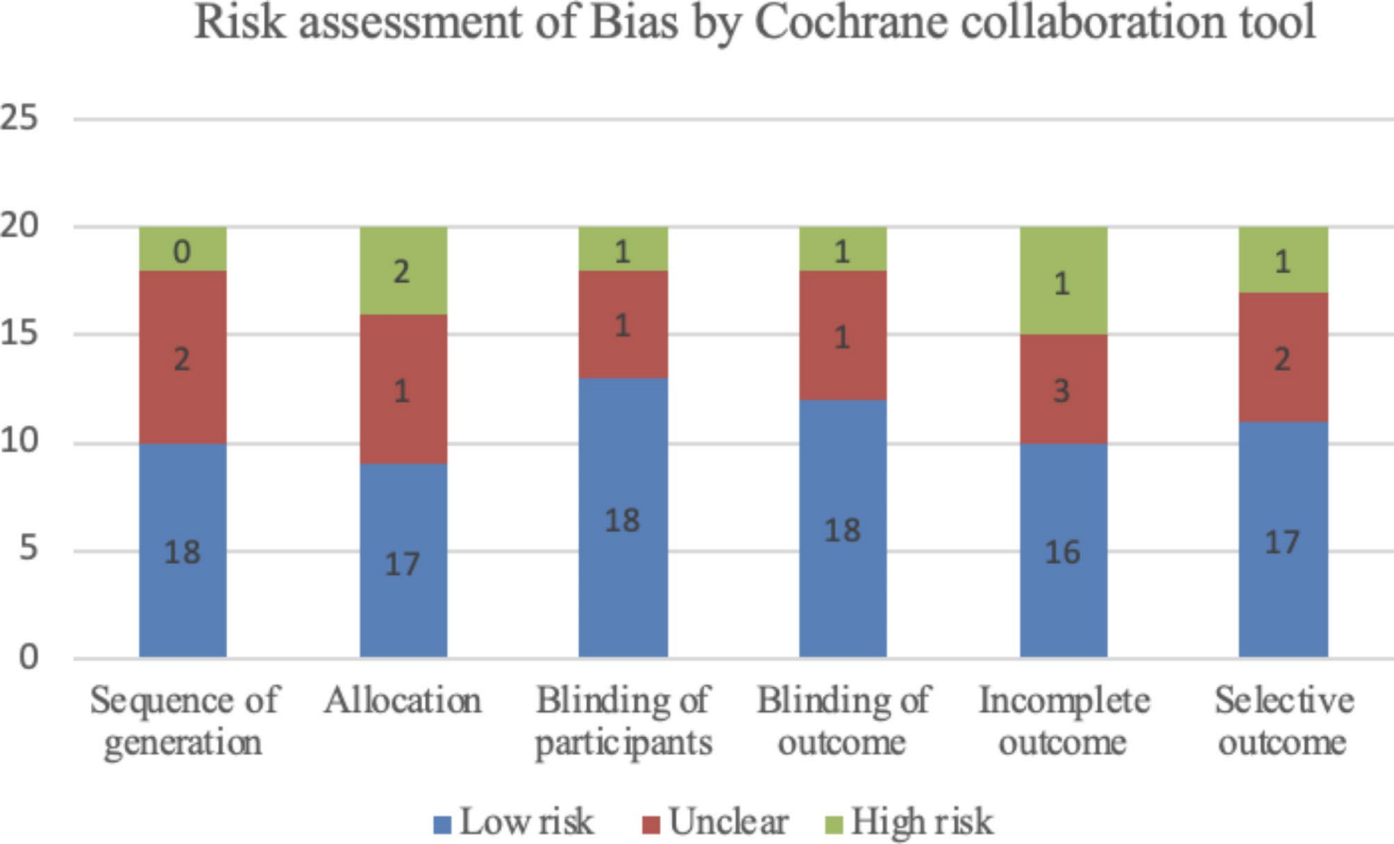
Risk assessment of bias by Cochrane collaboration tool

This systematic review utilized the Cochrane Risk of Bias Tool to evaluate any potential risks of included articles consisting of randomized clinical trials and ROB-ME tool (Risk Of Bias due to Missing Evidence in a synthesis) to evaluate any potential risks in included systematic reviews. This risk assessment tool enabled us to create papers of the highest caliber with solid conclusions. The following criteria were used to evaluate the subjective risk of bias in relevant articles and classify it into one of three levels based on factors like sequence generation, allocation concealment, participant blinding, blinding outcome, incomplete data outcome, and selective outcome reporting. There were approximately 86.66% low risk judgments, 8.33% equivocal judgments, and 5% high risk judgments, according to the Cochrane risk of bias assessment tool (Fig. 2).

A quality assessment using the CASP (Critical Appraisal Skill Program) scale checklist was done to determine the strengths and limitations of the chosen studies by evaluating their methodologies. All CASP checklists will check the validity, results and clinical relevance of included articles of this systematic review. By selecting yes, no, or not as an answer to each of the eleven questions posed, the validity, transparency, and standardization of the included studies were evaluated using the CASP scale. Table 2.

**Table 2:** Quality assessments done by the Critical Appraisal Skills Program scale (CASP) for selected studies of systematic review

Study No.	1	2	3	4	5	6	7	8	9	10	11	12	13	14	15	16	17	18	19	20
Clearly focused question	Y	Y	Y	Y	Y	Y	Y	Y	Y	Y	Y	Y	Y	Y	Y	Y	Y	Y	Y	Y
Appropriate design	Y	Y	Y	Y	Y	Y	Y	Y	Y	Y	Y	Y	Y	Y	Y	Y	Y	Y	Y	Y
Appropriate recruitment	Y	Y	Y	Y	Y	Y	Y	Y	Y	Y	Y	Y	Y	Y	Y	Y	Y	Y	Y	Y
Matched control	N/D	N/D	N/D	Y	Y	Y	Y	Y	Y	N/D	Y	Y	Y	Y	N/D	N/D	Y	Y	Y	Y
Test procedure clearly described	Y	Y	Y	Y	Y	Y	Y	Y	Y	Y	Y	Y	Y	Y	Y	Y	Y	Y	Y	Y
Appropriate outcomes used	Y	Y	Y	Y	Y	Y	Y	Y	Y	Y	Y	Y	Y	Y	Y	Y	Y	Y	Y	Y
Outcome accurately measured	Y	Y	Y	Y	Y	Y	Y	Y	Y	Y	Y	Y	Y	Y	Y	Y	Y	Y	Y	Y
Confounding factors accounted	N/D	N/D	N/D	Y	Y	Y	Y	Y	Y	N/D	Y	Y	Y	Y	N/D	N/D	Y	Y	Y	Y
Appropriate analysis	Y	Y	Y	Y	Y	Y	Y	Y	Y	Y	Y	Y	Y	Y	Y	Y	Y	Y	Y	Y
Precise statistical results	Y	Y	Y	Y	Y	Y	Y	Y	Y	Y	Y	Y	Y	Y	Y	Y	Y	Y	Y	Y
Interpretation of evidence	Y	Y	Y	Y	Y	Y	Y	Y	Y	Y	Y	Y	Y	Y	Y	Y	Y	Y	Y	Y

*Y-Yes; N-No; N/D- Not Determined

### Qualitative synthesis

Twenty research studies were subjected to qualitative synthesis, with key findings extracted from each study. The type of the study, type of clinical trial, the number of participants, intervention and the treatment outcomes are all taken from the data and summarized in Table 1.

## Discussion

To improve patient satisfaction levels in terms of aesthetics and restoring the architecture and function of lost tooth parts as a result of the disease, it is now crucial to understand the significance of periodontal phenotype and how it affects periodontal and restorative treatment outcomes [27–30].

The treatment results are influenced by the thickness of the gingival and bone tissues, possibly as a result of a differential in blood flow and the underlying bone’s susceptibility to resorption. Prior to beginning the restorative treatment, it is usually crucial to ascertain the periodontal tissue biotype. The relationship between GT and KTW been examined by various research studies. The majority of investigations generally discovered a favourable relationship between the maxillary anterior teeth KTW and GT. Several studies assessed the relationship between periodontal phenotype and gingival thickness. In most research evaluating maxillary anterior teeth, periodontal phenotype, GT, and KTW were found to be positively correlated with each other. The literature generally indicates that in the anterior maxilla, GT and periodontal phenotype are linked. There is little and inconsistent data available for locations other than the anterior maxilla [31–38].

Positive features of thick gingival tissue quality that have been documented in the literature include resistance to trauma and recession, superior soft tissue handling properties compared with thin tissue, promotion of creeping attachment, reduction in clinical inflammation, and improvement of predictable surgical outcomes. Thick soft tissue is thought to be able to survive because of its large extracellular matrix and collagen content as well as its improved vascularity [39–44].

The success of restorative dentistry greatly depends on the periodontal health, which is dependent on the restoration’s continuing integrity. According to research, the periodontal apparatus of teeth is extremely sensitive to even the smallest environmental changes, necessitating extraordinary caution to preserve periodontal health and avoid accidental harm. This demonstrates the unmistakable link between periodontal health and repair. Margin, contour, occlusion, material, bridge design, removable partial denture design, and restorative dental techniques are crucial factors from a periodontal perspective [45–48].

Using vestibular incision subperiosteal tunnel access (VISTA), In 2023, Min S. et al. conducted a research to explore the kinetics of tissue thickness increase as a phenotypic modification therapy measure following treatment of various gingival recession disorders. Multiple gingival recession abnormalities were successfully treated with VISTA and sub-epithelial connective graft (SCTG), which produced sustained improvements in gingival thickness and suggested a feasible technique for phenotypic modification [49, 50].

In 2020, Tatum CL, et al. undertook a study to examine the results of immediate single-implant insertion in individuals with thick or thin tissue morphologies in aesthetic areas. According to the study’s findings, immediate implant placement results for individuals with various soft tissue morphologies do not significantly differ from one another [51–56].

In 2021, Barootchi S. et al. published the results of a randomized clinical experiment that looked at how gingival phenotype changed after root coverage with freeze-dried or solvent-dehydrated acellular dermal matrix (FDADM and SDADM), respectively. The investigations came to the conclusion that after 9 years, the results of gingival phenotypic change were still present in both groups and across all sites [57].

Rasperini G, et al., conducted a research in 2020 to investigate the influence of gingival phenotype (GPh) upon the surgical outcome of coronally advanced flap (CAF). The study’s findings demonstrated that, when compared to individuals with medium, thick, and extremely thick GPh, patients with thin GPh had the lowest mean root coverage and total root coverage [58].

Ahmed AJ et al. released a research in 2018, to determine the effects of interdental papillae (PIP) presence on periodontal biotype (PB), papillary proportions (PP), the distance between facial and palatal papillae (DFPP), and the base of interproximal contact area to interproximal bone crest (CP-BC). The results of this study show that PB changed the proportion of papillae and the distance among the facial and palatal papillae, which influenced the heights and existence of maxillary interdental papillae [59–61].

In 2021, Beire JM, et al. did a study to assess the periodontal phenotype (PP) and its morphometric changes in dental students with healthy periodontal tissues using cone-beam computed tomography (CBCT). According to the study’s findings, thick PP predominated in the sample examined, and in all measurements tested, gingival thickness was always thinner than bone thickness [62–74].

Within the parameters of the current systematic review, the prevalence of different periodontal phenotypes is associated with successful periodontal and restorative treatment outcomes. Understanding the significance of periodontal phenotypes can be used to create aesthetic results for individuals who needed restorative and periodontal therapy operations.

The limitation of this study was that meta-analysis of the periodontal phenotype impact on restorative and periodontal treatment outcome was not possible due to the lack of a uniform number of journals.

## Conclusion

Practitioners’ knowledge of thin and thick periodontal phenotypes and the importance of periodontal phenotypes both in periodontology and restorative dentistry will help to generate outstanding esthetic results for patients who require restorative and periodontal therapies. Based on the findings gathered from this systematic review, individuals with narrow and thin gingiva typically exhibit higher rates of gingival recession in comparison to those with thick and wide gingiva. Positive correlations exist between gingival thickness, keratinized tissue, and bone morphotype and treatment outcomes in periodontics and restorative dentistry.

## Data Availability

The corresponding author will have access to the data that were the basis for this article.
